# Engagement With and Use of Health Information on Social Media Among US Latino Individuals: National Cross-Sectional Survey Study

**DOI:** 10.2196/59387

**Published:** 2025-02-24

**Authors:** Yonaira M Rivera, Kathryna Corpuz, Tahilin Sanchez Karver

**Affiliations:** 1 Department of Communication School of Communication and Information Rutgers University New Brunswick, NJ United States; 2 Office of Clinical and Health Affairs Rutgers Biomedical & Health Sciences New Brunswick, NJ United States; 3 Department of Health, Behavior & Society Johns Hopkins School of Public Health Baltimore, MD United States

**Keywords:** Latinos, health misinformation, engagement, utilization, social media, health information, United States, national, trends, survey, pandemic, non-Latino whites

## Abstract

**Background:**

During the COVID-19 pandemic, US Latino individuals were more likely to report accessing coronavirus information on social media than other groups, despite copious amounts of health misinformation documented on these platforms. Among the existing literature on factors associated with engagement and use of health information, racial minority status has been associated with greater susceptibility to health misinformation. However, literature to date has not reported national trends on how Latino individuals engage with or use health information on social media compared to non-Latino White (NLW) individuals, nor whether perceptions of the amount of health misinformation on social media influence health information engagement and usage.

**Objective:**

This study aimed to examine differences in engagement with and use of health information on social media among Latino and NLW individuals in the United States.

**Methods:**

We examined a nationally representative cross-sectional sample of Latino (n=827) and NLW (n=2563) respondents of the 2022 Health Information National Trends Survey who used social media in 2022 to assess differences in engagement with and use of health information. Items related to the perceived quantity of health misinformation on social media, social media use frequency, health information engagement (sharing content; watching videos), and health information usage (health decision-making; discussions with health care providers) were selected to conduct weighted bivariate analyses and logistic regressions.

**Results:**

Latino individuals perceive lower amounts of health misinformation on social media (28.9% perceived little to no misinformation vs 13.6% NLW individuals, *P*<.001). Latino audiences also reported higher health information engagement compared to NLW individuals (20% vs 10.2% shared information several times a month or more, *P*<.001; 42.4% vs 27.2% watched videos several times a month or more, *P*<.001), as well as higher information usage for health decision-making (22.8% vs 13.7%, *P*=.003). When controlling for ethnicity and other sociodemographic variables, perceiving lower amounts of health misinformation on social media was associated with higher odds of watching videos more frequently, making health decisions, and discussing health-related content with a health care provider (*P*<.001). Furthermore, Latino audiences were 1.85 times more likely to watch videos (*P*<.001), when controlling for the perceived amount of health misinformation and other sociodemographic variables. Finally, when compared to NLW individuals perceiving little to no health misinformation, Latino audiences perceiving little to no health misinformation were 2.91 times more likely to watch videos (*P*<.001).

**Conclusions:**

The findings suggest that Latino individuals engage with visual health (mis)information at higher rates. Digital health literacy interventions should consider video formats and preferred social media platforms among Latino individuals. Further research is warranted to understand sociocultural factors important to Latino social media users when consuming health information, as these may impact the success of digital media literacy interventions that teach users how to navigate misinformation online.

## Introduction

### Background

Social media’s ubiquitous presence in daily life has allowed for the democratization of health information and for individuals and communities to view, engage with, and share a variety of content with people in their networks. However, it has also contributed to the rise of health misinformation [1-4], which tends to evoke an emotional response and a reaction from its readers [5-7]. This drive to act can propel social media users to share viewed content with others without necessarily validating its credibility and integrate it into their own behaviors, including health-seeking behaviors. This can have a significant impact on their overall health outcomes [8], particularly in light of misinformation shared unintentionally to voice people’s concerns, find answers to their questions, and understand those answers in the context of their own life experiences [9]. Understanding the ramifications of misinformation in health outcomes, thus, requires an interdisciplinary approach that intersects the fields of health communication, communication and technology, and public health education.

### Social Media Usage and Health Information Consumption Among US Latino Individuals

Individuals identifying as Hispanic, Latino, Latina, or Latinx (henceforth Latino) are the largest and fastest growing minority group in the United States [10] and are also avid social media users [11]. In 2021, the Pew Research Center reported general social media usage rates among Latino adults in the United States, where 85% used YouTube, 72% used Facebook, 52% used Instagram, and 46% used WhatsApp [11]. YouTube (Google) has also been reported to be a top source for news content among Latino adults, particularly among those seeking content in Spanish: 48% of Latino households that predominantly speak Spanish rely on YouTube for news content compared to 36% among those that speak English [12]. Latino individuals also report a greater reliance on social media and messaging platforms than other groups, viewing platforms like WhatsApp, Instagram, and Facebook (Meta) as trustworthy tools to connect with families and friends in the United States and internationally [13]. This reliance on social media contributes to Latino individuals being at a higher risk for health misinformation exposure, as has been seen with topics like COVID-19, guns, and reproductive health matters including abortion [14]. For example, the National Association of Latino Elected and Appointed Officials’ 2022 National Latino Voter tracking poll reported that 76% of Latino survey respondents were exposed to news that abortion is now illegal in the United States and that individuals can be imprisoned for seeking abortion services [15]. Furthermore, Latino individuals may engage with content in English, Spanish, or a combination of both [13], adding another challenging layer in efforts to understand how these audiences engage with and use health information found on these platforms. Previous literature has highlighted inequities in Spanish-speaking Latino individuals’ ability to access and understand cancer information compared to English-speaking counterparts [16], yet studies exploring Spanish health content on social media are limited. This is particularly concerning given reports that platforms like Facebook have failed to flag misinformation in Spanish more so than misinformation in English, with one 2020 study reporting that an estimated 70% of Spanish-language misinformation on Facebook lacked warning labels [17].

Early during the COVID-19 pandemic, Latino individuals were reported to use social media to access COVID-19 information at higher rates than other groups in the United States [18]. However, given that social media platforms also contain user-generated content that is oftentimes not evidence-based, there is an increased likelihood for users to engage with and use health misinformation. For example, qualitative research exploring the Latino individuals’ engagement with cancer information on Facebook pre-COVID found that participants mainly engaged with content from potentially unreliable sources predominantly shared by Facebook friends [19]. Some participants also reported using encountered cancer information to discuss with friends and family and to implement new or reinforce current health behaviors, despite this content not always being evidence-based or reliable [19]. Despite these findings, the literature to date has not reported national trends in how Latino individuals engage with health information on social media compared to non-Latino White (NLW) individuals, nor whether the information they have engaged with has triggered offline actions that can influence health outcomes. As such, our first research question (RQ) asks “Does engagement with and usage of health information encountered on social media differ between Latino and NLW social media users?”

### Factors Associated With Health (Mis)Information Susceptibility and Their Role in Engagement and Usage

In an effort to better understand why some individuals may be more susceptible to health misinformation on social media, Nan and colleagues [20] conducted a systematic review of 61 studies to explore the psychological, demographic, and behavioral predictors of susceptibility. After presenting an integrative psychological model of susceptibility to health misinformation, authors found that psychological predictors included directionally motivated reasoning or actions driven by one’s emotions; placing greater trust in information shared by family, friends, politicians, or journalists; conservative or political ideology; and religiosity. Greater social media use was generally observed to increase one’s susceptibility to health misinformation, while demographic factors like age, education, sex at birth, and income showed mixed correlations to increased susceptibility to misinformation. The review also noted studies that suggest Latino individuals tend to be more susceptible [21-23], although none of these explicitly focused on this ethnic group.

One area that has received less attention relates to whether an individual’s perceptions of the amount of health misinformation that exists on social media has an impact on subsequent engagement and usage of that information. Chou et al [4] briefly bring attention to this issue in their 2020 commentary, “Where We Go From Here: Health Misinformation on Social Media,” which calls for research identifying the characteristics that make certain populations more vulnerable to the impacts of health misinformation. More recently, Goldsmith et al [24] conducted a systematic review exploring the use of social media as a source of COVID-19 information among migrant and ethnic minority populations, which found that some populations may rely on health misinformation to shape their understanding and inform their actions. The authors specifically referenced a study by Cervantes et al [25] that had a sample of 60 US Latino adults who were hospitalized for COVID-19. Many individuals in the study discussed using social media for COVID-19 recommendations, despite some acknowledging that COVID-19 misinformation was circulating at the time [25]. Similarly, Rivera et al [26] reported cases of Latino interviewees not verifying cancer-related information from Facebook because “someone must have” previously done so. Combined, these qualitative findings raise the question of whether differences in the Latino individuals’ perceptions about the amount of health misinformation on social media may impact engagement with and use of that content. It also begs the question of whether any such differences in perception impact engagement and usage outcomes differently than for NLW individuals. Therefore, we also ask the following:

RQ2. Do perceptions of the amount of health misinformation on social media differ between Latino and NLW social media users?

RQ3. Are perceptions of the amount of health misinformation on social media associated with engagement with and use of health information on social media upon controlling for social media usage and other sociodemographic variables?

RQ4. Does engagement with and use of encountered health information differ between Latino and NLW social media users who perceive the same amount of health misinformation on social media, upon controlling for social media usage and other sociodemographic variables?

## Methods

### Overview

This paper analyzes data from the 2022 Health Information National Trends Survey (referred to as HINTS 6). HINTS is a national cross-sectional survey conducted annually by the National Cancer Institute to assess access and use of health information by the general adult public in the United States. HINTS 6 covers topics related to looking for health information; finding information online; health care access and infrastructures; general health prevention; cancer screening and awareness; cancer beliefs; and cancer history [27]. HINTS 6 was conducted via a self-administered web or paper survey from March to November 2022. An equal-probability sample of addresses provided by Marketing Systems Group was selected from within four sampling strata (urban and rural areas with high vs low concentrations of minority adults) [28]. A total of 6252 individuals participated in the survey (response rate=28.1%). Full sample selection details are available in the HINTS 6 Methodology Report [28]. In order to explore engagement with and use of health information encountered on social media among individuals identified as Latino or Hispanic compared to NLW individuals, the analyses in this paper focus on participants who identified their race/ethnicity in the survey and reported using social media (n=3390). Acknowledging the heterogeneity that exists in Latino communities in the United States, it is important to delineate that HINTS 6 defines Latino or Hispanic participants as anyone who answers “yes” to the question, “Are you of Hispanic, Latino/a, or Spanish origin?” This includes anyone living in the United States who identifies as being part of any Latino subethnicity, regardless of racial background.

### Measures

#### Dependent Variables

##### Engagement With Health-Related Information

Two items were selected to measure engagement with health-related information on social media: frequency of sharing general health–related information on social media and frequency of watching health-related videos on a social media site. These outcomes were assessed by survey items asking participants: “In the past 12 months, how often did you share general health-related information on social media (eg, a news article)?” and “In the past 12 months, how often did you watch a health-related video on a social media site (eg, YouTube)?” Response options for both items included 1=almost every day, 2=at least once a week, 3=a few times a month, 4=less than once a month, and 5=never. Exploratory data analyses reported skewed findings (mean scores for sharing information 4.48, SD 0.82, and mean scores for watching videos 3.93, SD 1.05). Therefore, final binary outcome variables were created by recoding each response option as 0=never to less than once a month and 1=a few times a month or more.

##### Use of Health-Related Information

Two items were selected to measure the use of encountered health-related information on social media: using information encountered on social media to make health decisions and discussing information encountered on social media with a health care provider. These outcomes were assessed by survey items asking participants how much they agreed or disagreed with the following statements: “I use information from social media to make decisions about my health” and “I use information from social media in discussions with my health care provider.” Response options for both items included 1=strongly agree, 2=somewhat agree, 3=somewhat disagree, and 4=strongly disagree. Exploratory data analyses reported skewed findings (mean scores for making decisions 3.49, SD 0.76, and mean scores for discussing with health providers 3.38, SD 0.84). Therefore, final binary outcome variables were created by recoding each response option as 0=disagree and 1=agree.

#### Independent Variable: Perception of the Quantity of Misinformation on Social Media

The main factor associated with engagement with and usage of social media health information was the perception of the amount of health misinformation on social media. To explore differences in perception between Latino and NLW individuals, we first identified all participants who responded to the question “How much of the health information that you see on social do you think is false or misleading?”. Response options included 1=a lot, 2=some, 3=a little, 4=none, and 5=I don’t use social media; any participant who stated they did not use social media was dropped from the analysis. The remaining responses were recoded as 0=a little or none, 1=some, and 2=a lot.

To further assess whether differences in perceptions of the amount of health misinformation on social media between Latino participants and NLW participants were associated with engagement with and usage of social media health information, responses were divided by ethnicity (Latino vs NLW individuals). To do so, a final variable was created with the following six groups: 0=NLW individuals perceiving little to no health misinformation on social media, 1=NLW individuals perceiving some health misinformation on social media, 2=NLW individuals perceiving a lot of misinformation on social media, 3=Latino individuals perceiving little to no health misinformation on social media, 4=Latino individuals perceiving some health misinformation on social media, and 5=Latino individuals perceiving a lot of misinformation on social media.

Other covariates included categorical variables for age, education, sex at birth, and social media usage frequency (refer to Table 1 for category options). Social media use frequency was assessed by an item asking, “In the past 12 months, how often did you visit a social media site?” Responses were recorded as 0=never, 1=less than once a month, 2=a few times a month, 3=at least once a week, and 4=almost every day.

**Table 1:** Characteristics of study sample, Latino/a individuals and non-Latino/a White individuals, and weighted percentages (n=3390). All values presented, except for n, are weighted.

Variables			Latino/a, Unweighted (n=827), Weighted %		NLW^a^, Unweighted (n=2563), Weighted %		*P* value^b^	
**Sex at birth, n (%)**								.74
	Male	287 (47.2)		1013 (48.5)				
	Female	533 (52.8)		1545 (51.5)				
**Age (year), n (%)**								<.001
	18-34	218 (40)		365 (24.2)				
	35-49	249 (31.6)		533 (25.7)				
	50-64	214 (20.3)		772 (30.2)				
	65-74	111 (6.3)		572 (12.9)				
	75+	29 (1.8)		309 (6.9)				
**Education, n (%)**								<.001
	Less than high school	94 (13.9)		77 (4.3)				
	High school	178 (27.8)		363 (19)				
	Some college	259 (38.5)		681 (37.3)				
	College graduate or more	294 (19.9)		1436 (39.4)				
**Household income, n (%)**								<.001
	Less than US $20,000	174 (17.8)		239 (10.7)				
	US $20,000 to less than US $35,000	138 (16.5)		267 (9.3)				
	US $35,000 to less than US $50,000	117 (12.4)		278 (9.9)				
	US $50,000 to less than US $75,000	131 (18.7)		432 (17)				
	US $75,000 or more	237 (34.6)		1227 (53)				
**Social media use frequency, n (%)**								.04
	Almost daily	567 (75.5)		1681 (67.8)				
	At least once a week	100 (8.4)		358 (13.5)				
	A few times a month	65 (8.3)		207 (8.3)				
	Less than once a month	38 (3.7)		134 (4.2)				
	Never	50 (4)		172 (6.3)				
**Perceived amount of health misinformation on social media, n (%)**								<.001
	A little or none	223 (28.9)		369 (13.6)				
	Some	352 (43.3)		1152 (46)				
	A lot	252 (27.8)		1042 (40.5)				

^a^NLW: non-Latino White.

^b^Based on Pearson chi-square statistic.

### Data Analysis

Data were analyzed using SPSS (version 28; IBM Corp). Any missing variable responses were dropped from the corresponding analyses. Sampling weights provided in the dataset were applied using Taylor Series for variance estimation [29]. Frequencies were calculated by comparing all independent variables by ethnic group (Latino: n=827; NLW: n=2563) using chi-square tests. Bivariate analyses were conducted comparing the effects of perceived amount of health misinformation on social media, ethnic group, age, sex at birth, and social media use frequency in the past 12 months on all four outcomes. This was followed by multivariate logistic regressions to assess whether the perceived amount of health misinformation on social media and ethnic groups was associated with each outcome. Only statistically significant covariates were included in logistic regressions. Adjusted odds ratios, confidence intervals, and their *P* values for the independent variables were reported, and the significance level of all analyses was set at α=.05.

## Results

Participants in this sample were predominantly NLW individuals (75.6%, n=2563), female (61.5%, n=2078), 35-64 years old (52.4%, n=1768), had a college degree or higher (51.2%, n=1730), and had incomes of US $50,000 or more (62.6%, n=2027). Weighted demographics are listed in Table 1. Compared to NLW participants, Latino participants were more likely to be younger (*P<*.001), less educated (*P*<.001), and have lower incomes (*P*<.001).

### Perceptions of the Amount of Health Misinformation on Social Media Among Latino Audiences Compared to NLW Audiences

Table 1 also reports how participants use and perceive the social media environment. There were statistically significant differences in social media use, with the Latino individuals reporting more almost daily social media use than NLW individuals (75.5% vs 67.8%; *P*=.04). Latino individuals were also more likely to perceive there was little to no false or misleading health information on social media compared to NLW individuals (28.9% vs 13.6%; *P*<.001).

### Differences in Engagement With and Use of Health Information Encountered on Social Media Between Latino and NLW Individuals

Overall, Latino individuals reported higher levels of engagement with health information on social media in the last 12 months compared to NLW individuals (Table 2). Compared to 10.2% of NLW individuals, 20% of Latino individuals shared general health information on social media several times a month or more (*P*<.001). They also watched health videos on social media several times a month or more at higher amounts than NLW individuals (42.4% vs 27.2%, *P*<.001). Upon controlling for the perceived amount of health misinformation, frequency of social media usage, age, sex at birth, and education (Table 3), Latino individuals were 1.85 times more likely to watch health videos several times a month or more than NLW individuals (CI 1.38-2.47). They were also 1.65 times more likely than NLW individuals to share general health information on social media several times a month or more; these findings were marginally significant (CI 0.97-2.80).

**Table 2:** Reported engagement with and usage of health information on social media by perceptions of the amount of health misinformation on social media, Latino/a versus non-Latino/a White (NLW) individuals, weighted percentages. All values presented, except for n, are weighted.

Outcome variables			Latino/a, Unweighted n (Weighted %)		NLW, Unweighted n (Weighted %)		*P* value^a^
**Engagement**							
	Shared general health information on social media several times a month or more	137 (20)		248 (10.2)		<.001	
	Watched health-related videos on social media several times a month or more	330 (42.4)		658 (27.2)		<.001	
**Usage**							
	Used information to make a health decision	160 (22.8)		335 (13.7)		.003	
	Discussed information with health care provider	189 (22)		479 (18.4)		.12	

^a^Based on Pearson chi-square statistic.

Latino individuals also reported higher levels of use of health information encountered on social media when compared to NLW individuals; specifically, 22.8% of Latino individuals reported using encountered health information for health decision-making, compared to 13.7% of NLW individuals (*P*=.003; Table 2). However, differences between Latino and NLW individuals were not statistically significant upon controlling for perceptions of the amount of health misinformation on social media, frequency of social media usage, age, sex at birth, and education (Table 4). No statistically significant differences in discussing health information encountered on social media with a health care provider were found.

**Table 3:** Weighted unadjusted and adjusted odds ratios of engagement with health information on social media by perceptions of the amount of health misinformation on social media, Latino/a individuals versus non-Latino/a White individuals. Weighted adjusted models include frequency of social media usage, age, sex at birth, and education.

				Shared				Watched		
				Unadjusted OR^a^ (95% CI)		Adjusted OR (95% CI)		Unadjusted OR (95% CI)		Adjusted OR (95% CI)
**Race or ethnicity**										
	**NLW^b^ (reference)**									
		Latino/a	2.21 (1.40-3.51)^c^		1.65 (0.97-2.80)^c^		1.97 (1.47-2.63)^b^		1.85 (1.38- 2.47)^b^	
**Perceived health misinformation**										
	**A lot (reference)**									
		Some	1.57 (1- 2.48)^c^		1.04 (0.62- 1.77)		1.49 (1.15- 1.93)^c^		1.65 (1.26- 2.15)^c^	
		A little to none	2.14 (1.41- 3.24)^b^		1.43 (0.91- 2.24)		2.052 (1.47- 2.86)^c^		2.15 (1.53- 3.02)^c^	

^a^OR: odds ratio.

^b^NLW: non-Latino White.

^c^*P*<.05.

**Table 4:** Weighted unadjusted and adjusted odds ratios of the use of health information on social media by perceptions of the amount of health misinformation on social media, Latino/a versus non-Latino/a White individuals. Weighted adjusted models include frequency of social media usage, age, sex at birth, and education.

			Decided			Discussed	
			Unadjusted OR^a^ (95% CI)	Adjusted OR (95% CI)	Unadjusted OR (95% CI)		Adjusted OR (95% CI)
**Race and ethnicity**							
	**NLW^b^ (reference)**						
		Latino/a	1.87 (1.23- 2.82)^c^	1.41 (0.88- 2.25)	1.25 (0.94- 1.66)		1.10 (0.79- 1.54)
**Perceived health misinformation**							
	**A lot (reference)**						
		Some	3.26 (2.20- 4.83)^c^	4.15 (2.59- 6.65)^c^	1.96 (1.3- 2.93)^c^		2.34 (1.61- 3.40)^c^
		A little to none	4.39 (2.59- 7.44)^c^	6.26 (3.95- 9.92)^c^	2.49 (1.67- 3.73)^c^		2.86 (1.97- 4.16)^c^

^a^OR: odds ratio.

^b^NLW: non-Latino White.

^c^*P*<.05.

### Perceptions of Quantity of Health Misinformation on Social Media as a Factor Associated With Information Engagement and Use by Ethnicity

As seen in Tables 3 and 4, perceptions of the amount of health misinformation on social media were associated with engagement with and usage of encountered health information on social media in 3 of the 4 models, when controlling for ethnicity and other covariates. Specifically, individuals who perceived little to no health misinformation on social media were 2.15 times more likely to watch videos several times a month or more compared to individuals who believed there is a lot of health misinformation on social media (CI 1.53-3.02; Table 3). They were also 6.26 times more likely to use encountered information to make health decisions (CI 3.95-9.92) and 2.86 times more likely to discuss encountered health information with a health care provider (CI 1.97-4.16) than individuals who believed there is a lot of health misinformation on social media (Table 4).

Table 5 reports differences in engagement and usage between the Latino and NLW individuals by perceived amount of health misinformation to assess differences in these outcomes between ethnic groups who perceive the same amount of health misinformation on social media. Compared to NLW individuals perceiving little to no health misinformation on social media, Latino individuals perceiving little to no health misinformation were 2.91 times more likely to watch videos (CI 1.69-5.03), controlling for social media use frequency, age, sex at birth, and education. They also reported marginally significant higher odds of using encountered information to make health decisions when compared to NLW individuals perceiving little to no health misinformation (adjusted odds ratio 1.87, CI 0.96-3.63).

**Table 5:** Weighted adjusted odds ratios of perceived amount of health misinformation and ethnicity (Latino/a vs non-Latino/a White individuals) on engagement and usage of health information. Weighted adjusted models include frequency of social media usage, age, sex at birth, and education.

Perceived amount of health misinformation on social media		Engagement		Usage	
		Shared, Adjusted OR^a^ (95% CI)	Watched, Adjusted OR (95% CI)	Decided, Adjusted OR (95% CI)	Discussed, Adjusted OR (95% CI)
**Models 1-4^b^**					
	Latino audiences perceiving little to no health misinformation	1.67 (0.82-3.40)	2.91 (1.69- 5.03)^c^	1.87 (0.96- 3.63)	0.88 (0.46- 1.66)
**Models 5-8^b^**					
	Latino audiences perceiving some health misinformation	1.51 (0.66- 3.46)	1.87 (1.22- 2.88)^c^	1.31 (0.66- 2.60)	1.12 (0.69- 1.82)
**Models 9-12^b^**					
	Latino audiences perceiving a lot of health misinformation	1.84 (0.81- 4.17)	1.24 (0.72- 2.12)	0.92 (0.44- 1.91)	1.44 (0.78- 2.66)

^a^OR: odds ratio.

^b^Reference group for each is non-Latino White individuals with the same perceived amount of health misinformation on social media.

^c^*P*<.05.

When comparing Latino audiences to NLW audiences perceiving some health misinformation on social media, there were only statistically significant differences in the amount of health videos watched, with Latino audiences being 1.87 times more likely to watch videos on social media than NLW audiences (CI 1.87-2.88). No differences in engagement and usage were found when comparing Latino audiences to NLW audiences who perceived there is a lot of health misinformation on social media.

## Discussion

### Principal Findings

Multiple calls to action in the disciplines of public health and health communication have detailed the importance of honing in on subsets of communities that are most vulnerable to the impacts of health misinformation, so as to better target resources for intervention [4,30]. This work provides insights to the engagement and usage patterns of social media users who perceive little to no health misinformation exists on social media, despite evidence to the contrary [1-3]. Our findings suggest that perceptions of the amount of health misinformation may be an important factor of engagement and usage. More importantly, Latino populations appear to have lower perceptions of the amount of health information on social media compared to NLW populations. In addition, Latino individuals who actively seek health advice on social media have been reported to be more likely to hear or believe at least one false health claim [14]. Coupled with higher social media usage patterns [11,13], these trends may place members of the Latino population at a higher disadvantage when navigating the media landscape. It is important that future work focus on digital health literacy tailored to these subgroups.

### Comparisons With Previous Work

A growing body of literature has specifically highlighted concerns about the amount of health misinformation the Latino population may be exposed to on social media through visual formats. Rivera and colleagues [19,26] found evidence of Latino individuals both engaging with cancer misinformation encountered on Facebook in predominantly visual formats (ie, videos and images) and using it to make health decisions, despite most of the content not being evidence-based. Similarly, COVID-19 vaccine narratives in video format were highlighted as being easily accessible and shareable among Latino audiences, particularly those available in Spanish and shared through closed network platforms like WhatsApp [31]. These studies are consistent with findings that the Latino population has higher odds of watching health-related videos multiple times a month. In fact, Latino audiences spend more time streaming video content than other groups in the United states, with YouTube being the most used streaming platform [32]. Given recent evidence of YouTube’s poor quality of health-related content [33,34], future endeavors should target multilingual visual forms of health misinformation.

According to the Kaiser Family Foundation, almost half of Latino adults in the United States report using social media to seek health advice and information at least once a week [14]. In light of this, two additional findings merit further discussion despite their marginal significance. First, we found that Latino audiences were more likely to share general health information with others on social media than NLW audiences. Latino individuals have been reported higher trust in media shared and recommended by their friends and family than from other sources [31], signaling a potential point of intervention in efforts to minimize the dissemination of health misinformation and its downstream impacts. We also found that Latino audiences who perceived that little to no health misinformation exists on social media were more likely to make decisions with health-related content found on these platforms. This merits further exploration, as Rivera et al [19] also identified extreme cases where individuals reported potentially harmful behaviors (such as canceling a mammogram) in response to breast cancer misinformation videos shared via Facebook. Their qualitative work illuminated the role of interpersonal relationships in enhancing (and sometimes superseding) engagement with culturally relevant cancer content that came from potentially unreliable online sources, highlighting the interrelated ways in which cultural values and message and source factors contribute to engagement with social media health information [19]. As a budding area of inquiry, future research should identify important sociocultural factors and critical thinking skills that may predict engagement with content on social media among individuals with low digital health literacy, so as to better design interventions that curtail its negative impacts.

### Limitations

Several limitations to this work must be acknowledged. First, there is no way to ascertain how often surveyed individuals actually encountered health misinformation on social media. HINTS 6 items only ask about overall social media health information engagement and usage, which may include different rates of exposure among different populations [35]. Similarly, perceptions of misinformation were only assessed by one item and relied on personal interpretations of what misinformation is—a term that may be unfamiliar to some or misrecognized by others. This study also failed to control for regional differences, which may impact trends. Finally, given the cross-sectional nature of the dataset, we cannot make causal inferences related to the predictive nature of the amount of health misinformation individuals perceive exists on social media and how they engage with and use this information. It may be that engagement and usage impact perceptions of the amount of health misinformation on social media, as has been suggested by Stimpson and Ortega [36]. Future studies should design surveys to better assess causality.

Despite initial attempts to explore differences in engagement and usage by Latino subethnic groups, sample size limitations did not allow for these comparisons. HINTS 6 reports Latino population subethnicities as Mexican, Puerto Rican, Cuban, and Other. While a preliminary look at these subgroups suggests that Puerto Ricans living in the continental United States have engagement and usage rates similar to NLW individuals, a larger sample is needed to explore these factors. Similarly, the HINTS 6 dataset no longer includes items asking language preferences, making it difficult to explore the role of language as an antecedent to health information engagement and use of social media. While it does include an item for the selected survey language, bilingual individuals who prefer Spanish are missed. Finally, while HINTS 6 collects information about social media use generally, there are no items that identify the specific social media platforms used by respondents. Given Latino audiences’ reliance on platforms that are less popular to other US populations (ie, WhatsApp) [37], future iterations of HINTS may consider including this measure in their survey.

Finally, it is important to note that, as has been delineated by scholars elsewhere [16,38], the HINTS dataset was not designed to examine differences within Latino subgroups, nor does it include questions that may provide important sociocultural factors at play within this heterogeneous community. These may include cultural values like “familismo,” “personalismo,” “respeto,” and “simpatía”–values oftentimes associated with Latin American collectivist identities [39]*.* As researchers centering their work on the Latino community, we have discussed some of these complexities elsewhere [19,40] and believe future work should explore the impact these may have on social media engagement. Furthermore, there may be other mechanisms impacting engagement with online information such as trust [38], patient engagement [41], and media literacy [42] that should be explored in future studies. Nonetheless, the findings reported in this paper lay a foundation for this important line of inquiry.

### Conclusions

Our findings present new considerations for social media interventions to adequately deliver evidence-based health information to Latino populations. It is evident that video formats may better reach Latino audiences, despite the poor validity of many health-related videos on platforms like YouTube [33]. This raises the importance of building the digital health literacy of individuals and populations to improve their perception of whether a specific video or other modality of health information is actually valid and coming from a trustworthy source that can be used for health decision-making. Interventions should also consider multilingual digital media literacy training tailored to the specific social media platforms used by Latino populations, teaching them how to navigate each platform’s unique features and how to use these to better assess content that may be false. This is particularly important in light of recent changes in social media platforms that call for the cancelation of fact-checking services and replacing these with user-generated fact-checking labels [43,44].

## Abbreviations

HINTS: Health Information National Trends Survey
NLW: non-Latino White
RQ: research question

## Data Availability

The datasets analyzed during this study are publicly available at the National Cancer Institute’s Health Information National Trends survey website.
